# Growth pattern of ductal carcinoma in situ (DCIS): a retrospective analysis based on mammographic findings

**DOI:** 10.1054/bjoc.2001.1877

**Published:** 2001-07

**Authors:** J Z Thomson, A J Evans, S E Pinder, H C Burrell, A R M Wilson, I O Ellis

**Affiliations:** Departments of Radiology and Pathology, Pathology, City Hospital, Nottingham, UK

**Keywords:** breast, mammography, ductal carcinoma in situ, diagnosis

## Abstract

The aim of this study was to obtain information concerning the direction and rates of growth of ductal carcinoma in situ (DCIS). The previous mammograms of 124 women diagnosed with DCIS were examined. If in retrospect calcifications were present on the previous examination, the exact size and position were recorded on both diagnostic and previous imaging. The rates of change and direction of change in extent of calcifications were calculated. 39 women with a diagnosis of DCIS in retrospect had calcifications visible on both their current and prior examinations; these formed the study group. For individual clusters of calcification, change occurred along an axis to the nipple at a mean of 5.5 mm y^−1^and along an axis at 90° to the nipple at 2.6 mm y^−1^. Increase in calcifications along the axis to the nipple occurred at 2.6 mm y^−1^toward and 2.8 mm y^−1^away from the nipple. Increase in the axis to the nipple occurred at 1.8 mm y^−1^for low grade, 4.2 mm y^−1^for intermediate grade and 7.1 mm y^−1^for high grade. DCIS growth along an axis to the nipple occurs at over twice the rate of growth in the other direction(s) and growth toward and away from the nipple occurred equally. Growth rates increased with increasing nuclear grade of DCIS. These results validate nuclear grading of DCIS. Additionally, the results suggest that increased importance should be placed on identifying the ‘nipple’ and ‘anti-nipple’ margins of DCIS represented by calcifications for both surgical excision and pathological scrutiny. © 2001 Cancer Research Compaign http://www.bjcancer.com


*British Journal of Cancer* (2001) **85**(2), 225–227
© 2001 Cancer Research Campaign
doi: 10.1054/ bjoc.2001.1877, available online at http://www.idealibrary.com on IDEAL®

http://www.bjcancer.com

### Growth pattern of ductal carcinoma in situ (DCIS): a retrospective analysis based on mammographic findings

**JZ Thomson[1], AJ Evans[1], SE Pinder[2], HC Burrell[1], ARM Wilson[1] and IO Ellis[2]**

Departments of Radiology[1] and Pathology[2], City Hospital, Nottingham, UK

**Summary** The aim of this study was to obtain information concerning the direction and rates of growth of ductal carcinoma in situ (DCIS). The previous mammograms of 124 women diagnosed with DCIS were examined. If in retrospect calcifications were present on the previous examination, the exact size and position were recorded on both diagnostic and previous imaging. The rates of change and direction of change in extent of calcifications were calculated. 39 women with a diagnosis of DCIS in retrospect had calcifications visible on both their current and prior examinations; these formed the study group. For individual clusters of calcification, change occurred along an axis to the nipple at a mean of 5.5 mm $y^{-1}$ and along an axis at 90° to the nipple at 2.6 mm $y^{-1}$. Increase in calcifications along the axis to the nipple occurred at 2.6 mm $y^{-1}$ toward and 2.8 mm $y^{-1}$ away from the nipple. Increase in the axis to the nipple occurred at 1.8 mm $y^{-1}$ for low grade, 4.2 mm $y^{-1}$ for intermediate grade and 7.1 mm $y^{-1}$ for high grade. DCIS growth along an axis to the nipple occurs at over twice the rate of growth in the other direction(s) and growth toward and away from the nipple occurred equally. Growth rates increased with increasing nuclear grade of DCIS. These results validate nuclear grading of DCIS. Additionally, the results suggest that increased importance should be placed on identifying the 'nipple' and 'anti-nipple' margins of DCIS represented by calcifications for both surgical excision and pathological scrutiny. © 2001 Cancer Research Compaign http://www.bjcancer.com

**Keywords**: breast; mammography; ductal carcinoma in situ; diagnosis

The introduction of mammographic screening has led to an increasing number of cases of pure ductal carcinoma in situ (DCIS) diagnosed. In 1997/1998 the National Health Service Breast Screening Programme (NHSBSP, 1995) results show DCIS rates of 23.9% (prevalent round) and 20.9% (incident rounds) (Patnick, 1999). This compares with a DCIS rate of only 5% in a symptomatic population (Smart et al, 1978). Screening women under 50 years of age is associated with even higher proportions of DCIS lesions than screening those over 50 years of age (Evans et al, 1997).

Microcalcification is the commonest radiological feature of DCIS, being found in 85–90% of cases. Review of previous mammograms of women diagnosed with DCIS often shows calcification (Evans et al, 1999). This subgroup of patients with DCIS and calcific changes present (in retrospect) on prior mammograms allows observation of how DCIS changes over time. In this study we aim to identify the direction of growth of DCIS – using change in extent of calcifications as a measure of growth and to see if DCIS growth rates correlate with histological grade.

Information regarding growth directions may have practical value when planning wide local excision of DCIS lesions – which is the current treatment of choice, while assessment of growth rates would enable validation of current DCIS histological grading systems.

### MATERIALS AND METHODS

Review of a database of women with a diagnosis of DCIS without invasion identified 124 women in whom previous mammograms were available. The mammographic features of the DCIS on both previous and diagnostic mammograms were recorded. If calcifications were present, the number of calcifications were recorded. The extent of the calcification cluster was recorded, recording the distance to the nipple, the cluster extent in the axis of the nipple, and the maximum cluster extent in an axis at 90° to the nipple axis (the larger of the measurement from the MLO or CC view – if available). The interval between the previous and diagnostic mammogram was also recorded, allowing growth rates per year to be calculated. The radiologist reading the mammograms knew the patient had DCIS but was unaware of the grade of DCIS present. The results were then correlated with the pathological nuclear grade of the DCIS present, using the NHSBSP recommendations (NHSBSP Pathology Reporting, 1995). The differences found between grades and the increases in the cluster extent were analysed for statistical significance using the Mann–Whitney U and Spearman Rank Correlation methods respectively.

### RESULTS

124 women diagnosed with DCIS without invasion had previous mammograms available for comparison. 105 (85%) were found as a result of screening, 19 (15%) were symptomatic. 9 (7%) patients had a normal mammogram, 115 (93%) had abnormal findings, 91% of whom had calcifications present. Overall 105 (85%) of the 124 patients with DCIS had calcifications present at mammography. Following review of their previous mammograms 43 (35%) had some abnormality present at the same site. 39 (31%) had calcifications on both their current and previous films. The time interval between current and previous mammograms in women

*Received 9 January 2001*
*Revised 2 April 2001*
*Accepted 5 April 2001*

*Correspondence to:* AJ Evans with calcification on both mammograms ranged from 0.7 years to 4.2 years. 12 (28%) had been assessed previously and deemed benign and 31 (72%) had been overlooked or disregarded at the time.

The 39 patients with calcifications present on their current and previous films constitute the study group. Of these patients 6 (15%), 10 (26%) and 23 (59%) had low, intermediate and high grade DCIS respectively. For individual clusters of calcification, the mean area of calcification increased at a rate of 1.91 $cm^2\ y^{-1}$. The clusters of calcification increased their size in the axis of the nipple and along an axis at 90° to the nipple by 5.5 mm $y^{-1}$ and 2.5 mm $y^{-1}$ respectively, this difference is significant ($P < 0.0001$). Along the axis of the nipple, mean growth toward the nipple occurred at 2.6 mm $y^{-1}$ and growth away from the nipple occurred at 2.8 mm $y^{-1}$. The mean number of calcifications increased at a rate of 11 elements/year.

When the results are assessed as a function of nuclear grade, there is a strong correlation with increasing growth rates and increasing nuclear grade. Growth occurred along an axis drawn to the nipple at 1.8, 4.2 and 7.1 mm $y^{-1}$ for low ($n = 6$), intermediate ($n = 10$) and high ($n = 23$) grade lesions respectively. Growth along an axis at 90° to the nipple axis occurred at –0.2, 1.6 and 3.7 mm $y^{-1}$ for low, intermediate and high grade lesions respectively. Increasing growth rate is significantly associated with increasing DCIS grade. Low and intermediate grades combined have a slower growth rate than high grade DCIS ($P < 0.05$).

### DISCUSSION

The introduction of both mass population screening and stereotactic core biopsy techniques has resulted in increasing numbers of patients diagnosed with DCIS without invasion.

There have been relatively few attempts to understand the pathological behaviour of DCIS. If DCIS was found to have a predictable growth pattern, this should influence therapeutic options and surgical management, hopefully resulting in decreased numbers of patients requiring re-excision of involved margins or conversion to mastectomy.

The most successful pathological models of DCIS to date have been based on 3-dimensional studies and giant pathological sections of breasts containing DCIS and/or invasive disease (Holland et al, 1990; Faverly et al, 1994; Mai et al, 2000). These studies suggest that DCIS is usually unifocal and contiguous, involving only one quadrant in 66% of cases (Holland et al, 1990) although, the complicated anatomical distribution of the ducts may make it appear multicentric. Similar findings were reported by a group from Ontario, Canada (Mai et al, 2000). In their study of 28 patients with DCIS all but 1 patient had a focus of invasive disease (less than 30 mm in size). They concluded that DCIS involves ducts and acini belonging to the same lactiferous system within one quadrant as they found (1) connections between different foci of DCIS, (2) that DCIS was located in the same areas of the same duct system on serial levels of the coronal sections and (3) an apparent fanning out of DCIS at the periphery of the breast, consistent with the pattern of branching of the main lactiferous ducts. Their findings support the notion that carcinoma is usually confined to a single duct system (Faverly et al, 1994; Love and Barsky, 1996). This may well explain the good success rates seen with segmental resection of tumours, as the entirety of the involved duct system is excised. In their results this group found that DCIS was frequently seen to extend centrally toward the nipple and to fan out peripherally in accordance with the duct system. DCIS was usually seen to spread continuously, particularly in cases of high grade tumours. On occasions, discontinuous spread was seen. The authors felt this could be explained by either a multicentric/multifocal phenomenon or possibly tumour implantation.

The above studies of DCIS are based on a static pathological assessment of the breast at one point in time. This study is essentially a study of the natural history of calcific DCIS over time. A significant correlation between lesion size based on radiologic appearances (in patients with calcific DCIS) and pathologic size of the resected lesion has been demonstrated (Tan et al, 2000). This validates our method of assessing growth and extent of calcific DCIS based on the mammographic findings.

Similar rates of growth of DCIS have been reported by a group from Michigan, USA (Carlson et al, 1999). They examined the diagnostic mammograms of women with screen-detected DCIS, recorded the maximum diameter of the DCIS on the medio-lateral or cranio-caudal views and recorded the time elapsed since their last screening mammogram. They found a direct relationship between DCIS size and the length of the screening interval; the longer the interval, the larger the DCIS. They concluded that the data supports yearly mammographic screening for DCIS detection and management. Their data indirectly provide information on growth rates. Analysis shows a mean growth rate of 13 mm $y^{-1}$, however, their data are skewed by a small number of patients with extensive lesions. When the median growth rates are calculated they show a growth rate of 6.8 mm $y^{-1}$, which is very similar to our rate of 5.5 mm $y^{-1}$. 68% of their patient group and 59% of our group had DCIS of high nuclear grade. A table summarizing their data is shown in Table 1.

**Table 1** Relationship between screening interval and DCIS size and nuclear grade (from Carlson et al, 1999)

| DCIS | Screening interval | | | |
|---|---|---|---|---|
| Characteristics | Prevalent ($n = 24$) | Annual ($n = 77$) | Biennial ($n = 38$) | Triennial ($n = 27$) |
| Mean size (cm) | 3.29 | 1.69 | 2.27 | 3.49 |
| Median size (cm) | 3.00 | 1.21 | 1.55 | 1.8 |
| High nuclear grade | 14 (58.3) | 37 (48.1) | 29 (76.3) | 21 (78) |
| Intermediate nuclear grade | 8 (33) | 25 (33) | 4 (11) | 3 (11) |
| Low nuclear grade | 2 (8) | 15 (20) | 5 (13) | 3 (11) |

© 2001 Cancer Research Campaign

The results of this current study support the findings of the anatomical dissections that DCIS growth occurs along the duct system (i.e. along an axis to the nipple). We have found that growth in this axis occurs equally toward and away from the nipple. Growth in the axis to the nipple occurred at over twice the rate of growth in the opposite direction (i.e. along the axis drawn at 90° to the nipple). It is possible that at least part of the growth occurring in this direction is due to enlargement of the breast segment by tumour-filled ducts. This was seen in the dissection studies. These findings suggest that more attention should be given at conservation surgery to the 'nipple' and 'anti-nipple' margins for either generous excisions or anatomical resections of involved duct systems. Also, these margins could be identified for pathologists to allow greater scrutiny for assessment of margins.

This study has shown that nuclear grade of DCIS correlates with growth of DCIS in both the nipple plane and a plane 90° to the nipple. This finding, along with previous studies assessing local recurrence after wide local excision (Lagios et al, 1989), and the grade of invasive breast cancer arising from DCIS lesions (Lampejo et al, 1994), confirms the validity of current DCIS grading systems. It should be noted that the grading system used by Lagios and Lampejo were different to the grading system used in this study which is based purely on nuclear grade.

In conclusion, these results conform to the theory of single duct system involvement by DCIS. They show that growth occurs predominantly along an axis toward and away from the nipple at equal rates. Growth in other directions is slower and may simply represent expansion of the duct system by the intraduct tumour. These results suggest that particular importance should be placed on identifying the 'nipple' and 'anti-nipple' margins for achieving both adequate surgical excision and pathological scrutiny of excision margins. Rates of calcification cluster growth increased with increasing grade of DCIS and this appears to validate nuclear grading of DCIS.

### REFERENCE

Carlson KL, Helvie MA, Roubidoux MA, Kleer CG, Oberman HA, Wilson TE, Pollack EW and Rochester AB (1999) Relationship between mammographic screening intervals and size and histology of ductal carcinoma in situ. *AJR* **172**: 313–317

Evans AJ, Wilson ARM, Burrell HC, Ellis IO and Pinder SE (1999) Mammographic features of ductal carcinoma in situ (DCIS) present on previous mammography. *Clinical Radiology* **54**: 644–646

Evans WP, Starr AL and Bennos ES (1997) Comparison of the relative incidence of impalpable invasive breast cancer and ductal carcinoma in situ cancers detected in patients older and younger than 50 years of age. *Radiology* **204**: 489–491

Faverly DR, Burgers L, Bult P and Holland R (1994) Three dimensional imaging of mammary ductal carcinoma in situ: clinical implications. *Semin Diag Pathol* **11**: 193–198

Holland R, Hendricks JH, Verbeek AL, Mravunac M, Schuurmans Stekhoven JH (1990) Extent, distribution and mammographic/histological correlates of breast ductal carcinoma in situ. *Lancet* **335**(8688): 519–522

Lagios MD, Margolin FR, Westdahl PR and Rose MR (1989) Mammographically detected duct carcinoma in situ, frequency of local recurrence following tylectomy and prognostic effect of nuclear grade on local recurrence. *Cancer* **63**: 618–624

Lampejo OT, Barnes DM, Smith P and Millis RR (1994) Evaluation of infiltrating ductal carcinomas with a DCIS component: correlation of the histological type of the in-situ component with the grade of the infiltrating component. *Semin Diag Pathol* **11**: 215–222

Love SM and Barsky SH (1996) Breast-duct endoscopy to study stages of cancerous breast disease. *Lancet* **34**: 997–999

Mai KT, Yazdi HM, Burns BF and Perkins DG (2000) Pattern of Distribution of Intraductal and Infiltrating Ductal Carcinoma: A three-dimensional Study Using Serial Coronal Giant Sections of the Breast. *Human Pathology* **31**(4): 464–474

NHSBSP (1995) *Pathology Reporting in Breast Cancer Screening (2nd Ed.)*. NHSBSP Publication No3. Patnick J (Ed). NHSBSP Review 1999. Sheffield

Smart C, Myers M and Gloecker L (1978) Implications from SEER data on breast cancer management. *Cancer* **41**: 787–789

Tan PH, Ho JT, Ng EH, Chiang GS, Low SC, Ng FC and Bay BH (2000) Pathologic-radiologic correlations in screen-detected ductal carcinoma in situ of the breast: findings of the Singapore breast screening project. *International Journal of Cancer* **90**(4): 231–236

© 2001 Cancer Research Campaign 

## References

[PDF_00242] Carlson K. L., Helvie M. A., Roubidoux M. A., Kleer C. G., Oberman H. A., Wilson T. E., Pollak E. W., Rochester A. B. (1999). Relationship between mammographic screening intervals and size and histology of ductal carcinoma in situ.. AJR Am J Roentgenol.

[PDF_00246] Evans A. J., Wilson A. R., Burrell H. C., Ellis I. O., Pinder S. E. (1999). Mammographic features of ductal carcinoma in situ (DCIS) present on previous mammography.. Clin Radiol.

[PDF_00249] Evans W. P., Starr A. L., Bennos E. S. (1997). Comparison of the relative incidence of impalpable invasive breast carcinoma and ductal carcinoma in situ in cancers detected in patients older and younger than 50 years of age.. Radiology.

[PDF_00253] Faverly D. R., Burgers L., Bult P., Holland R. (1994). Three dimensional imaging of mammary ductal carcinoma in situ: clinical implications.. Semin Diagn Pathol.

[PDF_00256] Holland R., Hendriks J. H., Vebeek A. L., Mravunac M., Schuurmans Stekhoven J. H. (1990). Extent, distribution, and mammographic/histological correlations of breast ductal carcinoma in situ.. Lancet.

[PDF_00259] Lagios M. D., Margolin F. R., Westdahl P. R., Rose M. R. (1989). Mammographically detected duct carcinoma in situ. Frequency of local recurrence following tylectomy and prognostic effect of nuclear grade on local recurrence.. Cancer.

[PDF_00263] Lampejo O. T., Barnes D. M., Smith P., Millis R. R. (1994). Evaluation of infiltrating ductal carcinomas with a DCIS component: correlation of the histologic type of the in situ component with grade of the infiltrating component.. Semin Diagn Pathol.

[PDF_00267] Love S. M., Barsky S. H. (1996). Breast-duct endoscopy to study stages of cancerous breast disease.. Lancet.

[PDF_00269] Mai K. T., Yazdi H. M., Burns B. F., Perkins D. G. (2000). Pattern of distribution of intraductal and infiltrating ductal carcinoma: a three-dimensional study using serial coronal giant sections of the breast.. Hum Pathol.

[PDF_00275] Smart C. R., Myers M. H., Gloeckler L. A. (1978). Implications from SEER data on breast cancer management.. Cancer.

[PDF_00278] Tan P. H., Ho J. T., Ng E. H., Chiang G. S., Low S. C., Ng F. C., Bay B. H. (2000). Pathologic-radiologic correlations in screen-detected ductal carcinoma in situ of the breast: findings of the Singapore breast screening project.. Int J Cancer.

